# Effects of omega-3 carboxylic acids on lipoprotein particles and other cardiovascular risk markers in high-risk statin-treated patients with residual hypertriglyceridemia: a randomized, controlled, double-blind trial

**DOI:** 10.1186/s12944-015-0100-8

**Published:** 2015-09-02

**Authors:** Richard L. Dunbar, Stephen J. Nicholls, Kevin C. Maki, Eli M. Roth, David G. Orloff, Danielle Curcio, Judith Johnson, Douglas Kling, Michael H. Davidson

**Affiliations:** Division of Translational Medicine & Human Genetics, Perelman School of Medicine at the University of Pennsylvania, 3600 Spruce Street, 8046 Maloney Building, Philadelphia, PA 19104-2699 USA; South Australian Health & Medical Research Institute, University of Adelaide, Adelaide, Australia; Midwest Center for Metabolic & Cardiovascular Research, Chicago, IL USA; Sterling Research Group, Cincinnati, OH USA; Medpace, Inc., Cincinnati, OH USA; Omthera Pharmaceuticals, Princeton, NJ USA; AstraZeneca, Wilmington, DE USA

**Keywords:** Hypertriglyceridemia, Omega-3 fatty acids, Triglycerides, Statin, Low-density lipoprotein cholesterol, Apolipoprotein CIII, Lipoprotein-associated phospholipase A_2_, Lipoprotein subclasses, Eicosapentaenoic acid, Docosahexaenoic acid

## Abstract

**Background:**

This study examined the effects of a mixture of highly bioavailable omega-3 carboxylic acids (OM3-CA) on nuclear magnetic resonance spectroscopy–assessed lipoprotein particle concentrations and sizes and other cardiovascular risk markers in statin-treated patients with fasting triglycerides (TG) ≥2.3 mmol/L (200 mg/dL) and <5.6 mmol/L (500 mg/dL) and at high cardiovascular risk.

**Methods:**

After a diet lead-in and statin-stabilization period, 647 patients were randomly assigned to receive capsules of control (olive oil, OO) 4 g/d, OM3-CA 2 g/d (plus OO 2 g/d), or OM3-CA 4 g/d for 6 weeks.

**Results:**

Compared with OO, low-density lipoprotein (LDL) particle size was increased with OM3-CA 2 g/d (*p* < 0.01) and 4 g/d (*p* < 0.001), and very low-density lipoprotein (VLDL) and high-density lipoprotein (HDL) particle sizes were decreased with both OM3-CA dosages vs. OO (*p* < 0.001 and *p* < 0.05 for VLDL and HDL, respectively). Total VLDL/chylomicron remnant particle concentration was reduced by 8.5 and 16.0 % with OM3-CA 2 and 4 g/d, respectively, vs. a 6.9 % reduction with OO (*p* < 0.001 for OM3-CA 4 g/d vs. OO). Total HDL particle concentration was also reduced by 1.5 and 3.2 % with OM3-CA 2 and 4 g/d, respectively, vs. a 0.6 % increase with OO (at least *p* < 0.05 for both comparisons). Changes in total LDL particle concentration were not significantly different for OO vs. OM3-CA at either dosage. Apolipoprotein (Apo) CIII levels decreased by 7.6 and 13.1 % with OM3-CA 2 and 4 g/d, respectively, vs. 3.2 % with OO (*p* < 0.001 for OM3-CA 4 g/d vs. OO). Lipoprotein-associated phospholipase A_2_ (Lp-PLA_2_) mass was reduced by 6.2 and 10.7 % with OM3-CA 2 and 4 g/d, respectively, vs. a 0.1 % increase with OO (*p* < 0.001 for both vs. OO). There were no significant differences between treatments in high-sensitivity C-reactive protein responses.

**Conclusion:**

OM3-CA were associated with shifts in lipoprotein particle sizes and concentrations, and reductions in Apo CIII and Lp-PLA_2_, in patients with hypertriglyceridemia while taking a statin.

**Trial registration:**

ClinicalTrials.gov Identifier NCT01408303.

## Background

The long-chain omega-3 polyunsaturated fatty acids eicosapentaenoic acid (EPA) and docosahexaenoic acid (DHA) have been used therapeutically as agents to treat hypertriglyceridemia [1]. A mixture of highly bioavailable omega-3 carboxylic acids (OM3-CA; Epanova®, AstraZeneca, Wilmington, DE, USA) [2] has been shown to significantly reduce triglycerides (TG) and non-high-density lipoprotein cholesterol (non-HDL-C) levels at dosages of 2 and 4 g/d when administered to patients with severe hypertriglyceridemia (≥5.6 mmol/L [500 mg/dL]) [3] and to statin-treated patients with TG ≥2.3 mmol/L (200 mg/dL) and <5.6 mmol/L (500 mg/dL) and at high risk for a cardiovascular disease (CVD) event [4].

In addition to their lipoprotein lipid–altering effects, omega-3 fatty acids reportedly affect lipoprotein particle subclass sizes and concentrations, including a shift toward an increased proportion of larger, buoyant LDL particles vs. smaller, denser, and potentially more atherogenic, LDL particles [5–7]. Omega-3 fatty acids have also been shown to reduce levels of apolipoprotein (Apo) CIII [4, 6, 7]. This is potentially important clinically because Apo CIII inhibits lipoprotein lipase activity and hepatic uptake of TG-rich lipoproteins [8–11], and Apo CIII associated with Apo B–containing particles is an independent predictor of CVD event risk [12]. Moreover, loss-of-function polymorphisms in the Apo CIII gene (*APOC3*) have been associated with lower TG and reduced coronary and ischemic vascular disease risks [13–15].

The present paper reports analyses from the Epanova combined with a Statin in Patients with hypertRiglycerIdemia to reduce non-HDL cholesTerol (ESPRIT) study, a randomized, controlled, double-blind, parallel-group, phase III clinical trial regarding the effects of OM3-CA at two dosages (2 and 4 g/d), compared with an olive oil (OO) control, on lipoprotein particle and subclass sizes and concentrations, as well as other cardiovascular risk markers, including circulating levels of Apo CIII, and the inflammatory markers lipoprotein-associated phospholipase A_2_ (Lp-PLA_2_) and high-sensitivity C-reactive protein (hs-CRP) [16], in high-CVD-risk, statin-treated patients with residual hypertriglyceridemia.

The primary results from this study, reported previously [4], showed that 2- and 4-g/d dosages of OM3-CA, compared with OO control, significantly lowered TG (14.6 and 20.6 %, respectively, vs. 5.9 %) and non-HDL-C (3.9 and 6.9 %, respectively, vs. 0.9 %) (all *p* < 0.05 or lower). Total cholesterol (total-C) and VLDL-C concentrations were also reduced vs. OO with both OM3-CA dosages, and the total-C/HDL-C ratio and Apo AI and Apo B levels were significantly reduced vs. OO with 4 g/d only (all at least *p* < 0.05). LDL-C significantly increased with OM3-CA 2 g/d (4.6 %), compared with OO (1.1 %; *p* = 0.025), but not with OM3-CA 4 g/d (1.3 %). Percent changes from baseline in HDL-C did not differ significantly between OO and either OM3-CA dosage group.

## Results

### Patients

As described previously [4], 647 patients were randomized — 216 to OO, 215 to OM3-CA 2 g/d (plus OO 2 g/d), and 216 to OM3-CA 4 g/d — and 623 patients completed the study. The results reported herein are from the 627 patients in the intent-to-treat population. There were no significant differences between treatment groups in demographic characteristics, statin use, or the intensity of the statins used. Patients were primarily non-Hispanic/non-Latino (82.6 %) and white (94.1 %) men (59.1 %) with a mean (standard deviation) age of 60.8 (9.6) years. Most of the patients used a statin alone (95.3 %), and the majority used low-intensity statins (56.0 %).

### Lipoprotein particle sizes and concentrations

Baseline, end-of-treatment, and LSM-BT percent change from baseline values for lipoprotein particle sizes and concentrations are presented in Table 1. The LDL particle size was significantly increased and the VLDL and HDL particle sizes were significantly reduced with both OM3-CA 2- and 4-g/d dosages vs. OO. Baseline and changes from baseline in LDL particle size according to the end-of-treatment TG concentration categories of <1.7 (150), 1.7–2.2 (150–199), and ≥2.3 (200) mmol/L (mg/dL), respectively, are shown in Table 2. There was a significant (*p* < 0.001) inverse correlation (Spearman’s rho) between changes in TG and LDL particle size in all treatment groups: OO −0.283, OM3-CA 2 g/d −0.244, and OM3-CA 4 g/d −0.242.

**Table 1:** Baseline, end-of-treatment, and percent change values for lipoprotein particle sizes and concentrations^a,b^

	Statin + OO	Statin + OM3-CA	Statin + OM3-CA
	4 g/d	2 g/d^c^	4 g/d
Variable	(*n* = 211)	(*n* = 209)	(*n* = 207)
Lipoprotein particle sizes			
VLDL (nm)			
Baseline	55.0 (5.7)	55.4 (5.6)	55.2 (5.9)
End-of-treatment	54.7 (5.9)	52.2 (5.4)	50.8 (5.8)
%Δ LSM-BT (95 % CI)	−0.6 (−1.7, 0.4)	−5.6 (−6.6, −4.6)^***^	−8.0 (−9.0, −7.0)^***^
LDL (nm)			
Baseline	19.9 (0.4)	19.9 (0.3)	19.9 (0.4)
End-of-treatment^d^	19.9 (0.4)	20.0 (0.4)	20.1 (0.4)
%Δ LSM-BT (95 % CI)	−0.1 (−0.2, 0.1)	0.3 (0.1, 0.5)^**^	0.6 (0.4, 0.8)^***^
HDL (nm)			
Baseline	8.5 (0.3)	8.5 (0.3)	8.5 (0.3)
End-of-treatment	8.5 (0.3)	8.4 (0.3)	8.4 (0.4)
%Δ LSM-BT (95 % CI)	−0.2 (−0.5, 0.1)	−0.6 (−0.9, −0.2)^*^	−0.5 (−0.9, −0.2)^*^
Lipoprotein particle concentrations			
Total VLDL/chylomicron remnant (nmol/L)			
Baseline	129 (47.0)	128 (43.0)	130 (48.2)
End-of-treatment	123 (53.4)	119 (47.0)	114 (54.4)
%Δ LSM-BT (95 % CI)	−6.9 (−11.1, −2.5)	−8.5 (−12.6, −4.1)	−16.0 (−19.8, −12.0)^**^
Large VLDL/chylomicron remnant (nmol/L)			
Baseline	13.2 (7.2)	14.0 (7.4)	13.9 (8.3)
End-of-treatment	12.7 (7.9)	9.6 (7.3)	7.5 (6.8)
%Δ LSM-BT (95 % CI)	−10.1 (−17.5, −2.0)	−36.8 (−42.0, −31.1)^***^	−55.8 (−59.5, −51.8)^***^
Medium VLDL (nmol/L)			
Baseline	72.1 (30.9)	69.7 (32.2)	72.0 (37.4)
End-of-treatment	68.4 (35.5)	62.9 (33.2)	60.0 (36.3)
%Δ LSM-BT (95 % CI)	−9.7 (−15.5, −3.5)	−12.6 (−18.2, −6.5)	−22.8 (−27.8, −17.4)^*^
Small VLDL (nmol/L)			
Baseline	43.5 (31.1)	44.3 (25.5)	44.2 (29.5)
End-of-treatment	42.4 (30.4)	46.8 (28.3)	46.8 (30.4)
%Δ LSM-BT (95 % CI)	−1.1 (−10.1, 8.7)	4.7 (−4.8, 15.3)	8.6 (−1.4, 19.6)
Total LDL (nmol/L)			
Baseline	1286 (360)	1309 (357)	1323 (342)
End-of-treatment	1317 (374)	1359 (391)	1359 (378)
%Δ LSM-BT (95 % CI)	2.0 (−0.6, 4.5)	3.3 (0.8, 6.0)	2.4 (−0.2, 5.0)
IDL (nmol/L)			
Baseline	74.9 (77.2)	79.0 (78.5)	75.5 (76.6)
End-of-treatment	75.6 (92.0)	72.9 (73.8)	70.7 (78.9)
%Δ LSM-BT (95 % CI)	−13.5 (−25.2, 0.2)	−6.9 (−19.7, 7.8)	−15.7 (−27.3, −2.3)
Large LDL (nmol/L)			
Baseline	661 (576)	660 (569)	656 (592)
End-of-treatment	389 (408)	439 (440)	445 (407)
%Δ LSM-BT (95 % CI)	−49.8 (−58.5, −39.3)	−40.1 (−50.6, −27.4)^***^	−21.6 (−35.4, −4.7)^***^
Small LDL (nmol/L)			
Baseline	1004 (310)	1014 (313)	1033 (296)
End-of-treatment	1024 (325)	1023 (331)	1008 (334)
%Δ LSM-BT (95 % CI)	1.0 (−2.7, 4.7)	0.3 (−3.3, 4.1)	−4.2 (−7.7, −0.7)^*^
Total HDL (mmol/L)			
Baseline	32.8 (5.8)	32.7 (6.2)	32.6 (6.8)
End-of-treatment	33.0 (6.1)	32.3 (6.1)	31.6 (6.7)
%Δ LSM-BT (95 % CI)	0.6 (−0.7, 1.9)	−1.5 (−2.8, −0.2)^*^	−3.2 (−4.5, −1.9)^***^
Large HDL (mmol/L)			
Baseline	2.6 (1.9)	2.4 (1.8)	2.4 (1.9)
End-of-treatment	2.6 (1.8)	2.2 (2.0)	2.2 (2.3)
%Δ LSM-BT (95 % CI)	−6.9 (−20.9, 9.5)	−39.0 (−48.2, −28.1)^***^	−29.0 (−39.8, −16.3)^***^
Medium HDL (mmol/L)			
Baseline	6.0 (3.2)	6.1 (3.4)	6.5 (5.1)
End-of-treatment	5.9 (3.4)	6.3 (3.3)	6.7 (3.5)
%Δ LSM-BT (95 % CI)	−6.6 (−12.2, −0.7)	3.9 (−2.4, 10.5)^*^	11.3 (4.6, 18.4)^***^
Small HDL (mmol/L)			
Baseline	24.1 (4.7)	24.2 (4.5)	23.7 (4.9)
End-of-treatment	24.5 (5.0)	23.7 (4.8)	22.7 (4.6)
%Δ LSM-BT (95 % CI)	1.7 (−1.0, 4.4)	−1.9 (−4.5, 0.7)^**^	−3.6 (−6.1, −1.0)^***^

Abbreviations: *%∆* percent change, *CI* confidence interval, *HDL* high-density lipoprotein, IDL = intermediate-density lipoprotein, *LDL* low-density lipoprotein, *LSM-BT* least-squares means–back transformed, *OM3-CA* omega-3 carboxylic acids, *OO* Olive oil, *VLDL* very low-density lipoprotein

^a^Means (standard deviation) are presented for baseline and end-of-treatment values

^b^Superscript letters represent p-values for each OM3-CA dosage vs. OO: ^*^ *p* < 0.05, ^**^ *p* < 0.01, and ^***^ *p* < 0.001

^c^Patients in the OM3-CA 2 g/d treatment arm also received OO capsules at a dosage of 2 g/d

^d^Number of patients with data in the OM3-CA 2 g/d treatment group = 208

**Table 2:** Baseline and change in low-density lipoprotein particle size by end-of-treatment triglyceride category

End-of-treatment TG category and	Statin + OO	Statin + OM3-CA	Statin + OM3-CA
LDL particle size	4 g/d	2 g/d^a^	4 g/d
	Mean (95 % CI)		
TG <1.7 mmol/L (<150 mg/dL)	(*n* = 15)	(*n* = 14)	(*n* = 33)
Baseline LDL particle size (nm)	20.33 (20.09, 20.57)	20.10 (19.89, 20.31)	20.17 (20.03, 20.31)
Δ from baseline LDL particle size (nm)	0.22 (0.03, 0.41)	0.15 (0.00, 0.30)	0.31 (0.17, 0.45)
TG 1.7–2.2 mmol/L (150–199 mg/dL)	(*n* = 33)	(*n* = 52)	(*n* = 54)
Baseline LDL particle size (nm)	20.12 (19.96, 20.28)	20.03 (19.95, 20.11)	20.05 (19.93, 20.17)
Δ from baseline LDL particle size (nm)	0.09 (−0.03, 0.21)	0.10 (0.04, 0.16)	0.17 (0.09, 0.25)
TG ≥2.3 mmol/L (≥200 mg/dL)	(*n* = 163)	(*n* = 142)	(*n* = 120)
Baseline LDL particle size (nm)	19.87 (19.82, 19.92)	19.89 (19.83, 19.95)	19.82 (19.77, 19.87)
Δ from baseline LDL particle size (nm)	−0.05 (−0.09, −0.01)	0.05 (0.00, 0.10)	0.04 (0.00, 0.08)

Abbreviations: *∆* change, *CI* confidence interval, *LDL* low-density lipoprotein, *OM3-CA* omega-3 carboxylic acids, *OO* olive oil, *TG* triglycerides

^a^Patients in the OM3-CA 2 g/d treatment arm also received OO capsules at a dosage of 2 g/d

OM3-CA lowered the concentration of VLDL/chylomicron remnants, but only the OM3-CA 4 g/d response reached statistical significance vs. OO. Large VLDL/chylomicron remnant particles were reduced significantly vs. OO in both OM3-CA groups, medium VLDL particles were reduced significantly vs. OO only in the 4 g/d group, and no significant differences between groups were observed for the small VLDL particle concentration response.

Responses in total LDL and intermediate-density lipoprotein (IDL) particle concentrations did not differ significantly between treatments. The concentration of large LDL particles was reduced markedly from baseline in the OO group and reduced to a significantly smaller extent in both of the OM3-CA groups. Small LDL particle concentration was relatively unchanged from baseline in the OO and OM3-CA 2 g/d groups, whereas there was a small decline in the OM3-CA 4 g/d group that reached statistical significance, compared with the OO response.

Total HDL particle concentration declined statistically significantly, compared with OO in both OM3-CA dosage groups. Reductions in large and small HDL particle concentrations contributed to these effects, as both were reduced, compared with the responses in the OO group, whereas medium HDL particle concentration increased in both OM3-CA dosage groups vs. OO.

### Apo CIII, Lp-PLA_2_, and hs-CRP

**Fig. 1:**
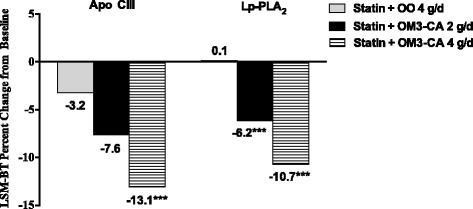
Percent changes from baseline in apolipoprotein CIII and lipoprotein-associated phospholipase A_2_ concentrations. Patients in the statin + OM3-CA 2 g/d group also received OO control at a dosage of 2 g/d. Superscript asterisks represent a significant difference vs. OO control (*p* < 0.001). Abbreviations: Apo = apolipoprotein, Lp-PLA_2_ = lipoprotein-associated phospholipase A_2_, LSM-BT = least-squares means–back transformed, OM3-CA = omega-3 carboxylic acids, OO = olive oil

Apo CIII decreased from baseline to end-of-treatment mean (standard deviation) concentrations of 15.0 (3.9) to 14.7 (4.1) mg/dL in the OO group, from 15.3 (3.9) to 14.2 (3.9) mg/dL in the OM3-CA 2 g/d group, and from 15.5 (4.0) to 13.4 (3.8) mg/dL in the OM3-CA 4 g/d group. Lp-PLA_2_ concentrations changed from 214 (50.2) to 215 (53.3) ng/mL, from 218 (54.7) to 205 (52.5) ng/mL, and from 216 (50.1) to 194 (51.4) ng/mL in the OO, OM3-CA 2 g/d, and OM3-CA 4 g/d groups, respectively. LSM-BT percent changes from baseline in Apo CIII and Lp-PLA_2_ concentrations are shown in Fig. 1. The reduction from baseline in Apo CIII concentration was significantly greater with OM3-CA 4 g/d vs. OO, but the comparison of the OM3-CA 2 g/d vs. OO responses did not reach statistical significance. Lp-PLA_2_ was significantly reduced from baseline with both the OM3-CA 2- and 4-g/d dosages, compared with the OO response. Mean (standard deviation) hs-CRP concentrations in the OO, OM3-CA 2 g/d, and OM3-CA 4 g/d groups, respectively, changed from 4.2 (5.7) to 4.1 (5.5) mg/L, a LSM-BT reduction of 2.8 %; from 3.9 (6.1) to 3.5 (4.2) mg/L, a 1.2 % increase; and from 4.2 (5.1) to 4.1 (6.1) mg/L, a 4.6 % reduction. LSM-BT percent changes from baseline in hs-CRP did not differ significantly between treatments.

## Discussion

In men and women with persistent hypertriglyceridemia while taking a statin, OM3-CA (a mixture of highly bioavailable omega-3 carboxylic acids whose main components are EPA, DHA, and dososapentaenoic acid) reduced mean VLDL/chylomicron particle size and concentration, increased mean LDL particle size without altering LDL particle concentration, and reduced mean HDL particle size and concentration. OM3-CA therapy also reduced circulating levels of Apo CIII and Lp-PLA_2_, but did not change hs-CRP concentration.

In an examination of 4.0 g/d of omega-3 acid ethyl esters (mainly EPA + DHA) in combination with simvastatin 20 mg/d, compared with placebo + simvastatin administered to patients with mixed dyslipidemia, VLDL particle size and concentration decreased, and LDL particle size significantly increased (all *p* < 0.05) without a change in LDL particle concentration [5]. Similarly, when the dosage of simvastatin was 40 mg/d, compared with placebo, EPA + DHA ethyl esters reduced mean VLDL particle size and increased LDL particle size [6]. The total VLDL and LDL particle concentrations were not altered by omega-3 treatment, relative to placebo (corn oil), but large VLDL and IDL particle concentrations were lowered and large LDL particle concentration was increased [6]. A study in mixed dyslipidemia of lipoprotein particle size and concentration changes with atorvastatin in combination with omega-3 acid ethyl esters vs. atorvastatin with placebo also demonstrated a mean increase in LDL particle size accompanied by a reduction in small LDL particle concentration and an increase in large LDL particle concentration [7].

The control-corrected increase in large LDL particle concentration in the 4.0 g/d OM3-CA group in the present study was similar to that observed in other studies with comparable dosages of omega-3 ethyl ester therapy in statin-treated patients [6, 7], despite higher baseline levels of large LDL particles in the present trial. The baseline small LDL particle concentration was lower in the current study than that in other similar trials [6, 7]. A modest increase in small LDL particle concentration was observed, whereas reductions occurred in the other trials [6, 7]. However, it should be noted that the on-treatment value for small LDL particles in the 4.0 g/d OM3-CA group remained below the on-treatment levels in other trials [6, 7]. Accordingly, the authors believe that the most likely explanation for the difference between studies in small LDL particle responses was the lower pre-treatment small LDL particle concentration in the current trial.

Some, but not all, studies have shown increases in LDL-C with omega-3 fatty acids therapy in patients with high or very high TG [17–19]. This effect appears to be attributable to DHA, as EPA alone has not been shown to raise LDL-C [20, 21]. The increased cholesterol is probably the result of an increase in mean LDL particle size, because trials in mixed dyslipidemia have consistently shown no increase in LDL particle concentration [5, 7]. In the present study, the results of the exploratory analysis of the relationship between LDL particle size and TG changes demonstrate that on-treatment TG level is an important determinant of the change in LDL particle size, and provide further support for the proposed threshold phenomenon, wherein TG must drop below a threshold for conversion from small, dense to larger, more buoyant LDL particles [6, 22, 23]. This TG threshold is specific to each individual, but is usually within the range of 1.1–2.8 mmol/L (100–250 mg/dL) [6, 22, 23].

Studies of prescription omega-3 acid ethyl ester therapy in statin-treated patients have shown that HDL-C is either increased modestly or unchanged [5, 6]; however, the concentration of HDL particles (as well as Apo AI) consistently declines [5, 6], as was observed in the present trial. The decrease in HDL particle concentration with omega-3 therapy is often accompanied by a modest elevation in HDL-C, which may reflect an increase in the quantity of cholesterol carried per HDL particle. Results from animal models suggest that fish oil enhances the cholesterol efflux capacity of HDL [24, 25]. Proteome changes with omega-3 therapy also support a potential improvement in HDL functionality, although enhanced cholesterol efflux capacity (a measure of HDL functionality) with omega-3 therapy in humans has not been demonstrated to date [26]. If confirmed in humans, this would have potential clinical relevance, because cholesterol efflux capacity has been found to be a better predictor of CVD risk than the HDL-C concentration [27]. At present, the clinical importance of changes in HDL-C, HDL particle and HDL particle subclass levels with omega-3 therapy are uncertain.

The present study also adds further evidence that omega-3 therapy lowers circulating concentrations of Apo CIII and Lp-PLA_2_. Elevated levels of both Apo CIII [8, 9, 11, 12] and Lp-PLA_2_ [28–30] have been associated with increased CVD event risk beyond that attributable to traditional risk factors. Previous studies with 4 g/d of EPA + DHA ethyl esters have shown statistically significant reductions of 11.5 % to 13.1 % in Apo CIII when added to statin therapy [6, 7]. Apo CIII in the current study was reduced by 7.6 % and 13.1 % with OM3-CA 2- and 4-g/d dosages, respectively, compared with a reduction of 3.2 % in the OO group (*p* < 0.001 for OM4-CA 4 g/d vs. OO).

The role that Apo CIII plays in the pathogenesis of hypertriglyceridemia is becoming increasingly evident [9, 10, 31]. Apo CIII inhibits the actions of lipoprotein lipase and hepatic lipase, thereby slowing TG hydrolysis, and it interferes with the interaction between TG-rich lipoproteins and hepatic Apo B/E receptors, further slowing TG removal from circulation [32–34]. The severity of hypertriglyceridemia is positively associated with the level of Apo CIII [9]. It has been suggested that an increase in Apo CIII synthesis in hypertriglyceridemia may represent a compensatory mechanism to reduce the catabolism of TG-rich lipoproteins and uptake by hepatic receptors in an attempt to cope with a large influx of substrates for TG production. In the present study, it was not possible to determine whether the reduction in Apo CIII was attributable to reduced production, increased clearance from circulation, or some combination. Regardless, it is likely that reducing Apo CIII with omega-3 fatty acid treatment contributes to TG lowering by enhancing the rate of TG clearance and/or reducing hepatic VLDL-TG secretion [35–37], and that Apo CIII may therefore also play a role in the non-HDL-C–lowering effect of OM3-CA.

Previous studies with 4 g/d of EPA + DHA ethyl esters have shown statistically significant reductions of 5.1 to 11.0 % in Lp-PLA_2_ when added to statin therapy [6, 7]. In the present study, Lp-PLA_2_ was reduced by 6.2 % and 10.7 % with OM3-CA 2 and 4 g/d, respectively, compared with a change of +0.1 % in the OO group (*p* < 0.001 for both dosages vs. OO). Of interest is that fenofibrate and niacin increase Lp-PLA_2_ when added to a statin [38, 39]. Omega-3 fatty acid treatment is the only lipid-altering therapy identified to date that lowers Lp-PLA_2_ mass without lowering LDL-C. Lp-PLA_2_ in circulation is a marker for secretion of PLA_2_ by macrophages in the arterial wall in response to inflammatory stimuli [40]. The clinical relevance of a reduction in Lp-PLA_2_ is uncertain at present, particularly in light of results from two clinical outcomes trials that failed to demonstrate reduced CVD event risk with darapladib, an oral, selective inhibitor of the Lp-PLA_2_ enzyme [41, 42]. As with Apo CIII, it is not possible in the present study to determine whether reduced Lp-PLA_2_ in response to omega-3 therapy resulted from reduced secretion of PLA_2_, reduced association of PLA_2_ with lipoproteins, or enhanced removal from circulation.

hs-CRP is an inflammatory marker, and an elevated level is associated with increased CVD risk [16, 43]. The present results affirm that OM3-CA does not significantly alter hs-CRP levels, which is consistent with results from other studies that have reported no effect of omega-3 fatty acid therapy on hs-CRP levels in statin-treated hypertriglyceridemic individuals [6, 7].

Although the results herein demonstrate that OM3-CA result in potentially favorable changes in lipoprotein particle concentrations and sizes, Apo CIII, and Lp-PLA_2_, similar to those shown for ethyl ester forms [5–7], they are limited in that they do not provide a mechanistic explanation for the changes observed, nor do they measure the impact on CVD risk. An Outcomes Study to Assess STatin Residual Risk Reduction with EpaNova in HiGh Cardiovascular Risk PatienTs with Hypertriglyceridemia (STRENGTH) is ongoing and will answer the question of the effects of OM3-CA on CVD risk (http://clinicaltrials.gov/show/NCT02104817). Another potential limitation that has been described previously [4] was the use of an OO control, which may have had non-neutral effects on some of the outcome variables. The control in the STRENGTH trial is corn oil.

## Conclusions

In men and women with persistent hypertriglyceridemia while taking statins, OM3-CA (a mixture of highly bioavailable omega-3 carboxylic acids whose major components are EPA, DHA, and docosapentaenoic acid) reduced the concentrations and sizes of VLDL/chylomicron remnant and HDL particles and increased LDL particle size without affecting LDL particle concentration. In addition, OM3-CA therapy reduced circulating levels of Apo CIII and Lp-PLA_2_, but did not change hs-CRP concentration. The potentially favorable changes in LDL particle size, Apo CIII, and Lp-PLA_2_ might contribute to reduced CVD risk with OM3-CA therapy; however, this remains to be demonstrated in clinical outcomes trials.

## Methods

### Study design and patients

ESPRIT was a trial conducted at 96 research sites in the United States. A list of the principal investigators and investigative sites is included in the Appendix. The full details of the study design and patient inclusion and exclusion criteria were published previously [4]. In brief, patients underwent a 6-week statin stabilization/National Cholesterol Education Program (NCEP) Therapeutic Lifestyle Changes diet lead-in period [44], after which those who met the eligibility criteria were randomized in approximately equal numbers to receive either OO 4 g/d, OM3-CA 2 g/d plus OO 2 g/d, or OM3-CA 4 g/d for 6 weeks in combination with the same dose of statin they were taking during the lead-in period. Good Clinical Practice Guidelines, the Declaration of Helsinki (2000), and the United States 21 Code of Federal Regulations were followed in the conduct of the study. An appropriately constituted Institutional Review Board approved the clinical protocol before the study started. All patients signed an informed consent form and provided authorization for disclosure of protected health information before undergoing any protocol-specific procedures.

Subjects included in the study were men and non-pregnant, non-lactating women ≥18 years of age with fasting TG ≥2.3 mmol/L (200 mg/dL) and <5.6 mmol/L (500 mg/dL) (after the statin/diet lead-in), at high risk for a future cardiovascular event, and at or near the NCEP goal for low-density lipoprotein cholesterol (LDL-C) (≤110 % of NCEP Adult Treatment Panel III LDL-C goal) [44] or on a maximally tolerated statin dose (stable for at least 4 weeks prior to screening). Disallowed agents included fish oil or any EPA- or DHA-containing products, medications, or investigational drugs within 6 weeks before randomization; and fibrates, bile acid sequestrants, niacin and its analogues >200 mg/d, simvastatin 80 mg, or any dietary supplement for the purpose of cholesterol lowering at screening or during the study. Subjects were instructed to follow the Therapeutic Lifestyle Changes diet and continue their routine activity levels throughout the study.

Exclusion criteria included a non-HDL-C level <2.3 mmol/L (90 mg/dL); known lipoprotein lipase impairment or deficiency, Apo CII deficiency, or familial dysbetalipoproteinemia; history of pancreatitis; type 1 diabetes mellitus, use of insulin, or glycated hemoglobin >10 %; poorly controlled hypertension; recent significant nephrotic syndrome or pulmonary, hepatic, biliary, gastrointestinal, or immunologic disease; cancer (except non-melanoma skin cancer or carcinoma *in situ* of the cervix); or clinically important clinical laboratory values at screening.

### Laboratory methods

Lipoprotein particle subclass concentrations and sizes were analyzed by LipoScience, Inc. (Raleigh, NC, USA), using the Nuclear Magnetic Resonance LipoProfile® method [45] on serum obtained from fasting (9–14 hr) blood samples collected at weeks −1 and 0 (values were averaged to calculate baseline) and weeks 5 and 6 of treatment (values were averaged to calculate end of treatment). Analyses of Apo CIII, Lp-PLA_2_, and hs-CRP were performed by Medpace Reference Laboratories (Cincinnati, OH, USA) on serum obtained from fasting serum samples collected at week 0 (baseline) and week 6 (end of treatment). Apo CIII concentrations were measured using the Randox Apo CIII test, which utilizes an *in vitro* turbidimetric immunoassay and the Randox Daytona analyzer (Kearneysville, WV, USA). Lp-PLA_2_ mass was determined by a latex particle–enhanced turbidimetric immunoassay on a Roche-P modular analyzer (PLAC™ test, Diadexus, San Francisco, CA, USA) [46]. hs-CRP was measured by nephelometry on a Siemens BNII nephelometer (Malvern, PA, USA).

### Statistical analyses

SAS (SAS Institute, Cary, NC, version 9.2) was used for statistical programming and analyses. End points included percent changes from baseline to end of treatment in lipoprotein particles (sizes, concentrations, and subclasses for very low-density lipoprotein [VLDL], LDL, and HDL), Apo CIII, Lp-PLA_2_, and hs-CRP. Efficacy end points for each OM3-CA arm were compared with OO using analysis of covariance, with the baseline value as a covariate and treatment group and statin intensity as factors. Low-intensity statins (defined as those expected to lower LDL-C by <40 % in patients with primary hyperlipidemia) included lovastatin 20–40 mg, pravastatin 10–80 mg, fluvastatin 20–80 mg, simvastatin 10–20 mg, atorvastatin 10–20 mg, and rosuvastatin 10 mg; high-intensity statins included simvastatin 40 mg, atorvastatin 40–80 mg, and rosuvastatin 20–40 mg [47, 48]. The response in each OM3-CA treatment group was compared to OO at a significance level of alpha = 0.05, two-sided, without adjustment for multiple comparisons. For patients who terminated participation prior to completing the full treatment period, the value of the previous post-randomization observation was carried forward. The Shapiro-Wilk test was run on the model residuals to investigate normality assumptions. Values were ranked prior to the final analysis if the normality assumption was rejected at *p* < 0.01. Because rank transformed data cannot be back-transformed into meaningful units, when they were used to generate p-values, models were also run using natural log-transformed values to produce least-squares means–back transformed (LSM-BT) and 95 % confidence intervals (CIs) for response values.

An exploratory analysis was performed that examined the baseline and changes from baseline in LDL particle size for patients according to their end-of-treatment TG category classified as <1.7 (150), 1.7–2.2 (150–199), or ≥2.3 (200) mmol/L (mg/dL) [44]. Univariate Spearman rank correlation coefficients within each treatment arm were calculated for the change from baseline LDL particle size as the dependent variable and change from baseline in TG concentration as the independent variable.

## Appendix

### Investigators and Clinical Sites

Yekaterina Khronusova, MD, Advanced Biomedical Research of America, Las Vegas, NV; Rebecca Jordan, MD, Center for Clinical Trials of Sacramento, Inc., Sacramento, CA; Jeffrey Rosen, MD, Clinical Research of South Florida, Coral Gables, FL; Samir Arora, MD, Columbus Clinical Research, Columbus, OH; Martha Hernandez-Illas, MD, Miami, FL; James Quigley, DO, Encinitas, CA; Michael Seidner, MD, Green and Seidner Family Practice Associates, Lansdale, PA; David Morin, MD, Holston Medical Group, Kingsport, TN; Cristian Breton, MD, International Research Associates, LLC, Miami, FL; Harold Bays, MD, Louisville, KY; Gigi Lefebvre, MD, St. Petersburg, FL; Evan Stein, MD, Metabolic and Atherosclerosis Research Center, Cincinnati, OH; Barry Lubin, MD, National Clinical Research – Norfolk, Inc., Norfolk, VA; James McKenney, PharmD, National Clinical Research – Richmond, Inc., Richmond, VA; Frederick Martin, MD, PMG Research of Bristol, LLC, Bristol, TN; Martin van Cleeff, MD, PMG Research of Raleigh, LLC, Cary, NC; George Raad, MD, PMG Research of Charlotte, LLC, Charlotte, NC; John Kessel, MD, PMG Research of Hickory, LLC, Hickory, NC; Bruce Boggs, MD, PMG Research of Johnson City, Gray, TN; John Rubino, MD, Raleigh, NC; Cecil Farrington, Jr., MD, PMG Research of Salisbury, Salisbury, NC; Jonathon Wilson, DO, PMG Research of Winston-Salem, Winston-Salem, NC; Andrea Lawless, MD, Biofortis Clinical Research, Addison, IL; David Oliver, DO, Renstar Medical Research, Ocala, FL; Eli Roth, MD, Sterling Research Group, Ltd., Cincinnati, OH; Dean Kereiakes, MD, The Carl and Edyth Lindner Center for Research and Education at The Christ Hospital, Cincinnati, OH; Wayne Harper, MD, Wake Research Associates, LLC, Raleigh, NC; Patricia Buchanan, MD, Williamette Valley Clinical Studies, Eugene, OR; Robert Weiss, MD, Maine Research Associates, Auburn, ME; Matthew Davis, MD, Rochester Clinical Research, Inc., Rochester, NY; Neil Fraser, MD, Troy Internal Medicine, PC Research Department, Troy, MI; Eric Klein, MD, Olympia, WA; Gary Levinson, MD, Integrated Research Center, Inc., San Diego, CA; Daniel Lorch, Jr., MD, PAB Clinical Research, Brandon, FL; Bradley Block, MD, Compass Research East, LLC, Oviedo, FL; Terry Klein, MD, Heartland Research Associates, LLC, Wichita, KS; Alan Forker, MD, Saint Luke’s Lipid and Diabetes Research Center, Kansas City, MO; Richard Glover, II, MD, Heartland Research Associates, LLC, Newton, KS; John Pullman, MD, Mercury Street Medical Group, PLLC, Butte, MT; Erich Schramm, MD, St. Johns Center for Clinical Research, Ponte Vedra, FL; Ronald Surowitz, DO, Health Awareness, Inc., Jupiter, FL; Craig Thompson, MD, Frederick C. Smith Clinic, Inc., Marion, OH; Phillip Toth, MD, Indianapolis, IN; Terence Hart, MD, Muscle Shoals, AL; Tami Helmer, MD, Radiant Research, Inc., Edina, MN; Kent Kamradt, MD/Michael Kyle, MD, Radiant Research, Inc., Chicago, IL; Marina Raikhel, MD, Lomita, CA; Venkata Challa, MD, High Point, NC; Dario Altamirano, DO, Hialeah, FL; Richard Beasley, MD, Health Concepts, Rapid City, SD; Bruce Bowling, MD, Regional Clinical Research, Inc., Endwell, NY; Alan Brown, MD, Oakbrook Terrace, IL; Lisa Cohen, DO, Suncoast Clinical Research, Inc., New Port Richey, FL; Craig Curtis, MD, Compass Research, LLC, Orlando, FL; Norman Fishman, MD, Diabetes & Endocrinology Specialists, Inc., Chesterfield, MO; David Hassman, DO, Comprehensive Clinical Research, Berlin, NJ; Timothy Koehler, DO, Heartland Research Associates, LLC, Wichita, KS; Audrey Lacour, MD, Houston, TX; David Larsen, MD, Wasatch Clinical Research, Salt Lake City, UT; Robert Lipetz, DO, Encompass Clinical Research, Spring Valley, CA; Richard Marple, MD, Castlerock Clinical Research Consultants, LLC, Tulsa, OK; Geri Poss, MD, San Antonio, TX; James Rhyne, MD, Statesville, NC; Jaime Sandoval, MD, Corpus Christi, TX; Deepica Reddy, MD, Westside Center for Clinical Research, Jacksonville, FL; Cynthia Strout, MD, Coastal Carolina Research Center, Mt. Pleasant, SC; Hugo Toro, MD, Houston, TX; Miguel Trevino, MD, Clearwater, FL; Kari Uusinarkaus, MD, Clinical Research Advantage, Inc./Colorado Springs Health Partners, PC, Tempe, AZ; Ralph Vicari, MD, Melbourne Internal Medicine Associates, Melbourne, FL; Carl Griffin, MD, Lynn Health Science Institute, Oklahoma City, OK; Larry Watkins, MD, Little Rock, AR; Ernie Riffer, MD, Clinical Research Advantage, Inc./Central Phoenix Medical Clinic, LLC, Tempe, AZ; Andrew Lewin, MD, National Research Institute, Los Angeles, CA; Terence Isakov, MD, Lyndhurst, OH; Bret Wittmer, MD, Commonwealth Biomedical Research, LLC, Madisonville, KY; Julie Mullen, DO/Albert Schreiner, III, MD, Sterling Research Group, Ltd., Cincinnati, OH; Gregory Gottsclich, MD, New Horizons Clinical Research, Cincinnati, OH; Brij Vaid, MD, Advance Clinical Research, St. Louis, MO; David Bolshoun, MD, Radiant Research, Inc., Denver, CO; Kenneth Lasseter, MD, Clinical Pharmacology of Miami, Inc., Miami, FL; Sashi Makam, MD, Mid Hudson Medical Research, PLLC, New Windsor, NY; Stephen Ong, MD, MD Medical Research, Oxon Hill, MD; Randall Severance, MD, Chandler, AZ; Michael Adams, MD, Radiant Research, Inc., Murray, UT; Richard Dunbar, MD, Penn-Presbyterian Medical Center, Philadelphia, PA; Richard Lorraine, MD, Harleysville, PA; Roger Miller, Jr., MD, Jacksonville, FL; Richard Mills, MD, Mount Pleasant, SC; Rajesh Patel, MD, Jersey Shore, PA; Irving Loh, MD, Westlake Medical Research, Westlake Village, CA; Clinton Corder, MD, COR Clinical Research, Oklahoma City, OK; Richard Dunbar, MD, Veterans Affairs Medical Center, Philadelphia, PA.

## Abbreviations

Apo: Apolipoprotein
CI: Confidence interval
CVD: Cardiovascular disease
DHA: Docosahexaenoic acid
EPA: Eicosapentaenoic acid
ESPRIT: Epanova combined with a Statin in Patients with hypertRiglycerIdemia to reduce non-HDL cholesTerol
HDL: High-density lipoprotein
HDL-C: High-density lipoprotein cholesterol
hs-CRP: High-sensitivity C-reactive protein
IDL: Intermediate-density lipoprotein
LDL: Low-density lipoprotein
LDL-C: Low-density lipoprotein cholesterol
Lp-PLA_2_: Lipoprotein-associated phospholipase A_2_
LSM-BT: Least-squares means–back transformed
NCEP: National Cholesterol Education Program
non-HDL-C: Non-high-density lipoprotein cholesterol
OM3-CA: Omega-3 carboxylic acids
OO: Olive oil
STRENGTH: A long-term outcomes Study to Assess STatin Residual Risk Reduction with EpaNova in HiGh Cardiovascular Risk PatienTs with Hypertriglyceridemia
TG: Triglycerides
total-C: Total cholesterol
VLDL: Very low-density lipoprotein
